# Knowledge of COVID-19 and the impact on indigents’ access to healthcare in Burkina Faso

**DOI:** 10.1186/s12939-022-01778-2

**Published:** 2022-10-27

**Authors:** E. Bonnet, Y. Beaugé, M. F. Ba, S. Sidibé, M. De Allegri, V. Ridde

**Affiliations:** 1 Institut de Recherche Pour Le Développement, UMR 215 PRODIG, 5, Cours Des Humanités, 93 322 Aubervilliers Cedex, France; 2 Heidelberg University, University Hospital and Medical Faculty, Heidelberg, Germany; 3 Institut de Santé Et de Développement (ISED), Cheikh Anta Diop University, Dakar, Senegal; 4 University Joseph Ki-Zerbo of Ouagadougou, Ouagadougou, Burkina Faso; 5 Institut de Recherche Pour Le Développement, Ceped, Université de Paris, Inserm ERL 1244, 45 Rue Des Saints-Pères, 75006 Paris, France; 6 Institut de Santé Et Développement, Université Cheikh Anta Diop, Dakar, Senegal

**Keywords:** Knowledge of COVID-19, Indigent, Access to healthcare, Low and middle-income countries

## Abstract

**Background:**

COVID-19 constitutes a global health emergency of unprecedented proportions. Preventive measures, however, have run up against certain difficulties in low and middle-income countries. This is the case in socially and geographically marginalized communities, which are excluded from information about preventive measures. This study contains a dual objective, i) to assess knowledge of COVID-19 and the preventive measures associated with it concerning indigents in the villages of Diebougou’s district in Burkina Faso. The aim is to understand if determinants of this understanding exist, and ii) to describe how their pathways to healthcare changed from 2019 to 2020 during the COVID-19 pandemic.

**Methods:**

The study was conducted in the Diebougou healthcare district, in the south-west region of Burkina Faso. We relied on a cross-sectional design and used data from the fourth round of a panel survey conducted among a sample of ultra-poor people that had been monitored since 2015. Data were collected in August 2020 and included a total of 259 ultra-poor people. A multivariate logistic regression to determine the factors associated with the respondents' knowledge of COVID-19 was used.

**Results:**

Half of indigents in the district said they had heard about COVID-19. Only 29% knew what the symptoms of the disease were. The majority claimed that they protected themselves from the virus by using preventive measures. This level of knowledge of the disease can be observed with no differences between the villages. Half of the indigents who expressed themselves agreed with government measures except for the closure of markets. An increase of over 11% can be seen in indigents without the opportunity for getting healthcare compared with before the pandemic.

**Conclusions:**

This research indicates that COVID-19 is partially known and that prevention measures are not universally understood. The study contributes to reducing the fragmentation of knowledge, in particular on vulnerable and marginalized populations. Results should be useful for future interventions for the control of epidemics that aim to leave no one behind.

## Background

Coronavirus disease 2019 (COVID-19) constitutes a global health emergency of unprecedented proportions [1]. Exceptional measures have been taken in different countries in an attempt to control the virus [2]. Preventive measures, however, have run up against certain difficulties in low and middle-income countries (LMICs) [3]. This is the case, most noticeably, in socially and geographically marginalized communities, which are often excluded from information about preventive measures [4], thus accentuating their vulnerability.

A large number of studies on populations’ knowledge and acceptance of the measures have been published worldwide [5, 6, 7]. Yet few studies focus on vulnerable and disadvantaged populations [8, 9], in Africa in particular. In a recent review on attitudes, knowledge and practices relating to COVID-19 in sub-Saharan Africa, there is not a single publication concerning these populations [10]. The state of knowledge is therefore piecemeal and centred around a global vision of the population without taking sufficient account of sub-groups in the populations as well as social health inequalities [11]. It is reasonable to assume that there are disparities in the social determinants of health relative to the general population, resulting in differential exposure to the virus, differential vulnerabilities to infection, and differential disease outcomes [12]. Furthermore, the question of these inequalities and the needs of the poorest are as often as not forgotten by those who formulate interventions in response to epidemics [13, 14].

These populations, and especially the poorest, namely the indigents, are often excluded from healthcare systems because of the cost of care, inadequate knowledge of their rights, or because they are unable to gain access to care [15, 16]. They are also likely to live far away from care services where little is known about their practices, in particular in times of crisis. Many studies, in effect, have been carried out in Africa, including in Burkina Faso, on knowledge and perceptions with regard to COVID-19 as well as recourse to care [10, 17, 18] but few studies focus solely on what occurred with the poorest. The methodological and ethical challenges to obtain data concerning this marginalized population are considerable but we have benefited from our work done on these populations over several years [19, 20] to gain a better understanding of their situation in the context of a pandemic.

Access to health services for the poorest and most marginalized populations is a historical challenge in Africa [21], and particularly in the Sahel where this study is based. Indeed, since the introduction of user charges at the end of the 1980s, people who are unable to pay face financial barriers to access that are often insurmountable [22]. These are in addition to non-financial barriers which are often significant, notably geographical distance, but also social exclusion [23]. In Burkina Faso, studies show that user fee exemption policies are not enough to improve the use of care by the indigents [24]. Furthermore, while most West African countries have user fees exemption policies for worst-off, these are often under-funded and not fully implemented as intended. In Burkina Faso, studies have shown that health professionals are not fully aware of and do not apply these policies for the most vulnerable; furthermore, as a result, the indigents frequently lack knowledge of their rights to this regard [25]. For example, when the COVID-19 pandemic spread, WHO proposed that countries should remove user fees at health facilities during this emergency. But a recent report from WHO Africa confirms that few countries have followed these recommendations, maintaining the financial barrier for marginalised populations [26].

At the start of the pandemic, many African states set up measures to halt the spread of SARS-CoV-2, often before the first case was even detected [27]. In Burkina Faso, these gradual measures began on 3 March 2020 with a ban on holding events on a national scale, the closure of educational establishments on 14 March, the suspension of prayers in places of worship and the introduction of a curfew, then border closures were introduced on 20 March, markets closed down on 24 March and travel between regions restricted on 26 March. In addition to these measures, there were national communication campaigns in every region on the ways to prevent the spread of the virus and explanations on the Coronavirus disease.

Official counts of COVID-19 cases and deaths have suggested moderate morbidity in sub-Saharan Africa. Difficulties in enumeration and reporting constraints at both local and central level have also contributed to underestimating the number of cases and deaths in these countries. Thus, several large-scale seroprevalence studies have been conducted in this region, revealing a much more considerable spread of SARS-CoV-2 [28] than what official statistics would suggest. In Burkina Faso, a study conducted in 2021 reported a seroprevalence of 50.6% in Bobo-Dioulasso and 32.6% in Ouagadougou. This research also confirmed the uniform spread of the virus throughout the country.

This study contains a dual objective, i) to assess knowledge of COVID-19 and the preventive measures associated with it concerning indigents in the villages and town of Diébougou in the health district of Diébougou in Burkina Faso. The aim is also to understand if determinants of this understanding exist, whether their socio-economic profile, proximity to healthcare centres, state of health have an impact on their understanding and ii) to describe how their pathways to healthcare changed from 2019 to 2020 during the COVID-19 pandemic.

This study dealt only with one healthcare district as it is incorporated within a wider research project on free healthcare for indigents in this region [29]. However, our thorough knowledge of the populations in this district enables us to envisage that the following findings will teach us a great deal about the impact of such a pandemic on these marginalized populations in general.

## Methods

### Conceptual framework

This study relied on the theory of the integrative model of health behaviour [30]. Our goal was to first assess the knowledge and preventive measures associated with COVID-19 among the indigent and then describe how their access to care has changed with the pandemic. Godin's integrative model guided our thinking about the range of variables to consider in understanding the indigents’ behaviour. The theory states that in order to build the model of health behaviour, it is necessary to consider several variables, grouped into three categories: 1) attitudes, 2) norms and 3) perceived control. Added to this are external variables that group individual characteristics (e.g., gender, age, socioeconomic status, education level, personality, etc.) and environmental variables.

### Study area and population

The study was conducted in the Diébougou healthcare district, in the south-west region of Burkina Faso. The district has a population of 139 824 inhabitants, over 40% of whom live under the threshold of poverty [31]. Diébougou has 24 public health establishments (4 dispensaries, 19 primary care establishments (CSPS: health and social promotion centres and a district hospital). There are 35 villages and the town of Diébougou.

In 2009, the Burkina Faso government published a decree providing free healthcare to the indigent. Its application was limited since checking the eligibility of indigent populations proved difficult. In 2014, a process of community-based targeting selected 6034 indigent people in the district [19, 20]. These indigents then received a card which gave them user fee exemptions for their care in the framework and duration of the PBF, which was implemented in eight of Burkina Faso’s healthcare districts. A random sampling among the poor, described elsewhere [29], made it possible to constitute the population for several research projects [19, 20] as well as that of the present study (*n* = 423), begun in 2020.

### The data and their sources

To answer study objective number one, we relied on a cross-sectional design and used data from the fourth round of a panel survey conducted among a sample of ultra-poor people that had been monitored since 2015 [19]. Data were collected in August 2020 and included a total of 259 ultra-poor people. Between 2015 and 2020, the number of people monitored went down (died or were lost sight of) which explains the variation in the number of indigents in the sample.

For study objective two, we relied on a pre–post design and used data from the third and the above-mentioned fourth round of the ultra-poor survey. Data for the third round were collected in June 2019 and reached a total of 292 respondents. The difference in sample size across the two survey rounds was due to the difficulties in monitoring the indigents over time.

The data collection tool was a structured, closed-ended questionnaire that included questions on: indigent identification, including village geolocation; sociodemographic and economic profile; exemption card (i.e., the card officially identifying the person as indigent and providing access to free health services under a pre-BPF scheme) and health service utilization; self-reported illness and perceived care needs; functional abilities and support network; and a COVID-19 section on knowledge of the disease (measures to protect oneself from the virus) and government measures.

The two rounds of surveys were conducted by five trained interviewers with local language skills under the supervision of a study coordinator. The interviewers visited the homes of the ultra-poor and sought their verbal consent to administer the questionnaire face-to-face in the local language. The interviewer was chosen according to the language spoken by the indigent to ensure that the questions were understood and to avoid confusion between COVID-19 and any other disease with similar symptoms. Tablets equipped with the Open Data Kit (ODK) software were used to administer the questionnaire, with the captured data transferred daily to the central database.

### Variables and their measurement

The outcome for objective 1 was a descriptive analysis of COVID-19 knowledge in indigents with the variables of sex, age, economic well-being quintile, educational level, marital status, good state of health and having a chronic disease. Concerning the variable relating to COVID-19, the symptoms of the disease (cough, runny nose, fever, fatigue), the protective measures (handwashing, mask wearing, not leaving the home, avoiding large gatherings), governmental measures (closure of markets and places of worship, curfew, travel between regions), as well as feeling anxious about the virus. Variables on the effectiveness of each measure were also put forward.

Concerning the analysis of the determinants of knowledge, the dependent variable, was knowledge about COVID-19, measured by the answer yes or no to the question “Have you heard about the new coronavirus disease (epidemic) called COVID-19?”. The independent variables were those mentioned above.

The variable of distance to the health centre was calculated in a GIS by measuring the Euclidian distance between the indigent’s living place and the nearest healthcare centre.

The outcomes for objective 2 were disease reporting, assessment of the search for healthcare and the total OOPE for those who used the health services.

Both objectives share the following common variables: age, which is a continuous variable. Sex (Male/Female) and having a chronic disease (Yes/No) are dichotomous variables. Marital status is a categorical variable comprising five modalities (single, in a monogamous union, in a polygamous union, widowed, divorced). The source variable was converted to a dichotomous variable (a married person and the other modalities). Self-reported health is a categorical variable (good, average, poor). The variable was converted to a dichotomous variable (Good / other modalities). The economic wellbeing quintile was obtained using Principal Component Analysis (PCA) on the ownership of sustainable assets and characteristics of the dwelling. This approach made it possible to classify the indigents from “poorest (1) to least poor” (5), in order to capture the socio-economic differences existing within indigence.

### Statistical analysis

We first described continuous variables by the mean ± standard deviation and the categorical variables by their frequencies for both samples 2019 (*N* = 292) and 2020 (*N* = 259).

Then, to pursue objective one, we used the 2020 sample and applied a chi-squared (Chi2) test to compare two categorical variables and a student’s t-test, a categorical variable and a continuous variable. Finally, we used multivariate logistic regression to determine the factors associated with the respondents' knowledge of COVID-19. The results are expressed as adjusted odds ratios (AOR). Significance was considered at a *p*-value < 0.05.

Then, to pursue objective two, we performed a comparative analysis to assess how the pathways to healthcare for the ultra-poor changed during COVID-19 from 2019 to 2020. In order to do so, we used both samples (2019 and 2020) and matched the observations (*N* = 220) at the individual level based on a unique identifier to receive a balanced panel. The characteristics of each study sample were assessed separately at baseline (2019) and endline (2020). The Chi2 and the student’s t-test were used to determine whether the baseline and follow-up samples had the same statistical distributions applying the same significance level as described above.

The geolocation data of indigents were incorporated in a Geographic Information System. Spatial analysis made it possible to calculate the distance separating each indigent from the nearest *CSPS*. We sought to assess if the proximity to a healthcare centre, designed to disseminate health prevention information, could explain improved knowledge of COVID-19.

Analyses were carried out using Stata version 16 software (Stata Corp, Lakeway Drive, College Station, TX, USA), R version 4.0.5 (R Foundation for Statistical Computing, Vienna, Austria) and ESRI ArcGis version 10.6 (ESRI Inc., Redlands, CA, USA).

## Ethics

The health ethics committee of Burkina Faso approved the study on 9 January 2019 (Decision No. 2019–01-004). The indigents took part in the study having given their informed verbal consent.

## Results

### Characteristics of the study population

Table 1 provides descriptive statistics, frequencies and percentages for the study samples 2019 (*N* = 292) and 2020 (*N* = 259) used to address the two above-mentioned objectives. The average age was 58.4 years in 2019 (56.9 years in 2020). The proportion of female indigents was 66.1% in 2019 (68.0% in 2020). The proportion of educated indigents was 9.9% in 2019 (6.9% in 2020). About 48% of the indigents were married, in both years. In the 2019 sample, 27.7% reported having a chronic disease (29.3% in 2020).

**Table 1:** Distribution of indigents according to the characteristics of the study population

Variable	**Sample 2019**		**Sample 2020**	
	***N* = 292**	**%**	***N* = 259**	**%**
Sex				
Female	193	66.1	176	68.0
Male	99	33.9	83	32.0
Education				
Uneducated	263	90.1	241	93.1
Educated	29	9.9	18	6.9
Maried:				
No	140	47.9	126	48.6
Yes	152	52.1	133	51.4
Good state of health				
No	204	69.9	175	67.6
Yes	88	30.1	84	32.4
Having a chronic disease				
No	211	72.3	183	70.7
Yes	81	27.7	76	29.3
Quintile				
Poorest	70	23.9	56	21.6
Poor	52	17.8	48	18.5
Average	55	18.8	53	20.5
Less poor	62	21.2	60	23.2
Least poor	53	18.1	42	16.2
	**Mean**	**Sd**	**Mean**	**Sd**
Age	58.4	17.8	56.9	19.0

### Characteristics linked to COVID-19

**Fig. 1:**
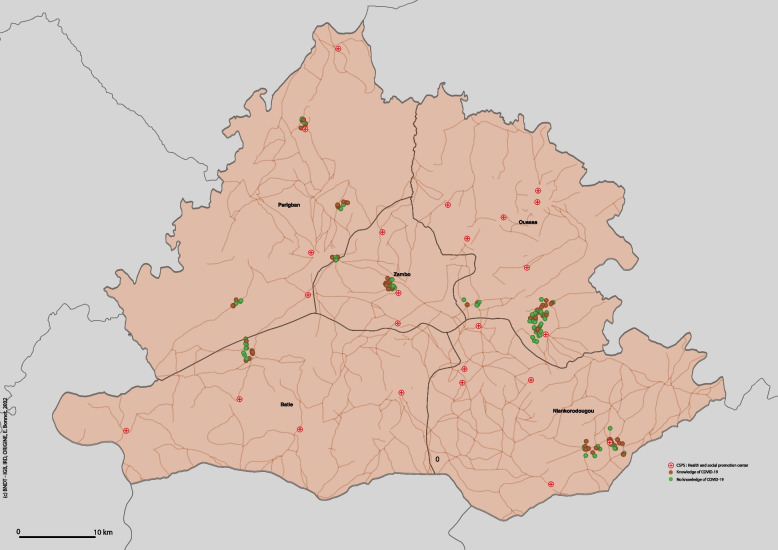
Indigents’ knowledge of COVID-19 in the district of Diébougou

With regard to the knowledge of COVID-19 and associated preventive measures of indigents in the health district of Diébougou, Burkina Faso (objective 1), the analysis showed that in total, 56% (*n* = 145) of the indigent respondents said they had heard of COVID-19 (Table 2). But 61% (= 89) of them said they did not know the symptoms of the disease. None of the symptoms of COVID-19 was known by over 30% of indigents except for a cough (32%). The red dots in Fig. 1 (Fig. 1) represent individual indigents with knowledge of COVID-19, while the green dots those with no knowledge. The spatial distribution of these points is random, and the distance between the indigents’ residence and the nearest health center is not associated with knowledge of COVID-19.

**Table 2:** Distribution of indigents’ responses according to the characteristics linked to COVID-19

Variable	**N**	**%**
Knowledge of COVID-19		
No	114	44.0
Yes	145	56.0
Fever		
No	114	78.6
Yes	31	21.4
Fatigue		
No	132	91.0
Yes	13	9.0
Cough		
No	99	68.3
Yes	46	31.7
Anxiety about COVID-19		
No	128	49.4
Yes	131	50.6
Avoiding going out		
No	50	34.5
Yes	95	65.5
More frequent handwashing		
No	103	71.0
Yes	42	29.0
Wearing a mask		
No	110	75.9
Yes	35	24.1
Avoiding gatherings		
No	120	82.8
Yes	25	17.2
Knowledge of government measures		
No	68	46.9
Yes	77	53.1
Knowledge of curfew		
No	22	28.6
Yes	55	71.4
Knowledge of ban on travel between regions		
No	25	32.5
Yes	52	67.5
Knowledge of closure of markets		
No	1	1.3
Yes	76	98.7
Knowledge of closure of places of worship		
No	27	35.1
Yes	50	64.9
Effectiveness of measures taken (curfew)		
No	12	21.8
Yes	43	78.2
Effectiveness of measures taken (ban on travel between regions)		
No	8	15.4
Yes	44	84.6
Effectiveness of measures taken (closure of markets)		
No	30	39.5
Yes	46	60.5
Effectiveness of measures taken (closure of places of worship)		
No	14	28.0
Yes	36	72.0

Among the indigents, 55% claimed they protected themselves against the virus. Out of these, 65% said they had avoided leaving home, but fewer than 30% said they washed their hands more often (29%), wore a mask (24%) or avoided gatherings (17%).

Only 35% were aware of anti-pandemic measures implemented by the government. Amongst these, it was the closure of markets which was the most well-known measure (98%), then the curfew (71%), the ban on travel between regions (67%) and the closure of places of worship (64%).

Only 56% (*n* = 145) of indigents spoke about the effectiveness of government measures to fight the pandemic. Among them, 77% (*n* = 76) agreed and strongly agreed with the implementation of a curfew, 84% recognized the effectiveness of banning travel between regions and 72% found that closing places of worship was an effective measure. Conversely, only 60% considered that closing markets was effective (Table 2).

Knowledge of COVID-19 in men was 66.3 whereas it was 51.1% in women (*p*-value = 0.031) (Table 3). There was no difference in knowledge according to the level of poverty. Conversely, almost all educated persons had knowledge of COVID-19 (94%) whereas only one out of two uneducated indigents (53%—*p*-value = 0,002) knew about the disease. Having received a card for user fee exemption for healthcare did not prove better knowledge of COVID-19 than others. On the contrary, 77% of those who had not received a card knew about COVID-19 compared with 53% who had (*p*-value = 0,027). There were fewer indigents who said they were in good health (46%) than those who said they did not enjoy good health (60%) who knew about COVID-19 (*p*-value = 0.044).

**Table 3:** Distribution of indigents according to the characteristics and knowledge of COVID-19

	**Knowledge of COVID-19**		
	**No**	**Yes**	***p*-value**
Sex:			**0.031**
Female	86 (48.9%)	90 (51.1%)	
Male	28 (33.7%)	55 (66.3%)	
Age			0.308
Under 25 years	15 (55.6%)	12 (44.4%)	
25 to 59 years	34 (39.1%)	53 (60.9%)	
60 years and over	65 (44.8%)	80 (55.2%)	
quintile:			**0.020**
Poorest	26 (46.4%)	30 (53.6%)	
Poor	31 (64.6%)	17 (35.4%)	
Average	20 (37.7%)	33 (62.3%)	
Less poor	22 (36.7%)	38 (63.3%)	
Least poor	15 (35.7%)	27 (64.3%)	
Education			**0.002**
Uneducated	113 (46.9%)	128 (53.1%)	
Educated	1 (5.6%)	17 (94.4%)	
Maried			1.000
No	55 (43.7%)	71 (56.3%)	
Yes	59 (44.4%)	74 (55.6%)	
Good state of health			**0.044**
No	69 (39.4%)	106 (60.6%)	
Yes	45 (53.6%)	39 (46.4%)	
Having a chronic illness			0.173
No	86 (47.0%)	97 (53.0%)	
Yes	28 (36.8%)	48 (63.2%)	

Table 4 shows the factors that are associated with knowledge of COVID-19 among the indigents. Men were more likely to be aware of COVID-19 than women. Educated indigents were more likely to be aware of COVID-19 than uneducated indigents. Finally, indigents with poor health status were more likely to experience COVID-19 than indigents with good health status.

**Table 4:** Results of the multivariate analysis

	**Model**		
	**AOR**	**95% CI**	***p*-value**
Sex:			
Female	**1**		
Male	**2.15**	**1.12, 4.21**	**0.023**
Age (µ ± σ)	1.00	0.98, 1.01	0.726
Quintile:			
Poorest	1		
Poor	0.45	0.18, 1.11	0.086
Average	1.70	0.74, 4.01	0.588
Less poor	1.34	0.59, 3.11	0.351
Least poor	1.15	0.45, 2.91	0.066
Education			
Uneducated	**1**		
Educated	**13.60**	**2.43, 258.00**	**0.015**
Maried			
No	1		
Yes	0.90	0.48, 1.68	0.745
Good state of health			
No	**1**		
Yes	**0.44**	**0.23, 0.83**	**0.012**
Having a chronic illness			
No	1		
Yes	1.31	0.71, 2.44	0.389

### Pathways to care

Turning towards how pathways to care changed from 2019 to 2020 (objective 2), we see that the only variable that significantly changed statistically was illness reporting (Table 5). Illness reporting by the ultra-poor decreased by 16.82% (*p* < 0.001). For the other variables of interest (conditional on illness reporting) such as reported health status, socio-economic status and the use of informal or formal health services, the results are inconclusive as they show a mixed picture and only statistically insignificant results which might also be due to the small sample size. We can only report trend percentages for these variables. From 2019 to 2020, the percentage of those who reported being in good health decreased by 3.60% (*p* = 0.42), the use of informal healthcare services conditional on illness reporting (e.g. traditional healers) went down by 3.17% (*p* = 0.56) and the total OOPE for those who sought any services whatsoever also went down by 1149.53 FCFA (*p* = 0.89). Meanwhile, a positive trend can be seen only in the use of formal healthcare services. Conditional on being ill, the use of formal health care services increased by 2.36% (*p* = 0.40).

**Table 5:** Changes in pathways to care for the ultra-poor from 2019 to 2020

	**2019 (*N* = 220)**	**2020 (*N* = 220)**	**Chi2 and T-test**
**Outcome**			
Illness reporting			** < 0.001**
No	83 (37.9%)	120 (54.6%)	
Yes	137 (62.1%)	100 (45.4%)	
Health service utilization (informal and formal) conditional on illness reporting			0.67
No	32 (23.4%)	21 (21.0%)	
Yes	105 (76.6%)	79 (79.0%)	
Health service utilization (formal) conditional on illness reporting			0.40
No	61 (44.5%)	39 (39.0%)	
Yes	76 (55.5%)	61 (61.0%)	
Health service utilization (informal) conditional on illness reporting			0.56
No	108 (78.8%)	82 (82.0%)	
Yes	29 (21.2%)	18 (18.0%)	
**Additional variables of interest conditional on illness reporting**			
Sex:			0.10
Female	92 (67.2%)	77 (77.0%)	
Male	45 (32.8%)	23 (23.0%)	
Quintile			0.85
Poorest	32 (23.4%)	26 (26.0%)	
Poor	24 (17.5%)	19 (19.0%)	
Average	25 (18.2%)	13 (13.0%)	
Less poor	30 (21.9%)	24 (24.0%)	
Least poor	26 (19.0%)	18 (18.0%)	
Education			
Uneducated			
Educated			
Married			0.39
No	68 (50.4%)	56 (56.0%)	
Yes	69 (49.6%)	44 (44.0%)	
Good state of health			0.42
No	117 (85.4%)	89 (89.0%)	
Yes	20 (14.6%)	11 (11.0%)	
Having a chronic illness			
No			
Yes			
Total OOPE Informal and formal for those who used services (µ ± σ)	13,698.9 ± 68,304.2	12,549.4 ± 24,676.2	0.89
**Outcome**			
Illness reporting			** < 0.001**
No	83 (37.9%)	120 (54.6%)	
Yes	137 (62.1%)	100 (45.4%)	
Health service utilization (informal and formal) conditional on illness reporting			0.67
No	32 (23.4%)	21 (21.0%)	
Yes	105 (76.6%)	79 (79.0%)	
Health service utilization (formal) conditional on illness reporting			0.40
No	61 (44.5%)	39 (39.0%)	
Yes	76 (55.5%)	61 (61.0%)	
Health service utilization (informal) conditional on illness reporting			0.56
No	108 (78.8%)	82 (82.0%)	
Yes	29 (21.2%)	18 (18.0%)	
**Additional variables of interest conditional on illness reporting**			
Sex:			0.10
Female	92 (67.2%)	77 (77.0%)	
Male	45 (32.8%)	23 (23.0%)	
Quintile			0.85
Poorest	32 (23.4%)	26 (26.0%)	
Poor	24 (17.5%)	19 (19.0%)	
Average	25 (18.2%)	13 (13.0%)	
Less poor	30 (21.9%)	24 (24.0%)	
Least poor	26 (19.0%)	18 (18.0%)	
Education			
Uneducated	127 (92.70%)	96 (96.00%)	0.29
Educated	10 (7.30%)	4 (4.00%)	
Married			0.39
No	69 (50.36%)	44 (44.00%)	
Yes	68 (49.64%)	56 (56.00%)	
Good state of health			0.42
No	117 (85.4%)	89 (89.00%)	
Yes	20 (14.6%)	11 (11.00%)	
Having a chronic illness			0.20
No	88 (64.23%)	56 (56.00%)	
Yes	44 (35.77%)	44 (44.00%)	
Total OOPE Informal and formal for those who used services (µ ± σ)	13,698.9 ± 68,304.2	12,549.4 ± 24,676.2	0.89

## Discussion

Almost half of indigents in the district said they had heard about COVID-19 but only 29% knew what the symptoms of the disease were. Yet the majority claimed that they protected themselves from the virus by using preventive measures. This level of knowledge of the disease can be observed with no noticeable differences between the villages. Half of the indigents who expressed themselves agreed with government measures except for the closure of markets, which represented for them the few places where they could socialize and obtain vital resources. Furthermore, an increase of over 11% can be seen in indigents without the opportunity for getting healthcare compared with before the pandemic.

This general analysis in the context of the COVID-19 pandemic, therefore, confirms that indigents are people who are isolated, far from sources of information and have had to suffer the consequences which reduced their recourse to health services. The study shows, in effect, that a large number of indigents had access to partial information on COVID-19 whereas national studies in Burkina Faso [32–34] show that 88% and 93% of the populations said they knew about the disease. A review focusing on Ethiopia, Nigeria, Cameroon, Uganda, Rwanda, Ghana, Democratic Republic of Congo, Sudan, and Sierra Leone [10] confirms that the populations, for the most part, had adequate declaratory behaviour of protection against COVID-19. In the northern centre of Nigeria, a study reports a very good knowledge of COVID-19 (99.5%) which is said to have been acquired mainly by Internet, social media and television [5] which we know are not tools used by indigents. Moreover, regarding the study area at Diébougou, not many targeted preventive actions were kept up following the first wave of the epidemic. Therefore, the knowledge of vulnerable and isolated populations could hardly be improved other than through regular access to radio and door-to-door campaigns as opposed to means used for other populations.

This general analysis, however, hides specific situations even within this vulnerable sub-group of the population [35]. Indeed, there are marked differences between indigents, since those who are more educated or enjoy better health have more opportunity to be informed about the disease and its risks. There are, therefore, inequalities even among the most vulnerable. The distance from healthcare centres does not seem to have an impact on these inequalities, doubtless because indigents as a whole use healthcare centres only infrequently, even when they benefit from free healthcare [36]. Furthermore, the lack of available research data concerning the pandemic in rural areas does not completely lead to an understanding of whether the pandemic had more effect on the poorest and the most isolated populations in rural areas. If the virus circulated throughout all the territories, testing facilities and identification do not make it possible to know those who were infected [27]. Testing centres are only located in the district capitals and are inaccessible to most populations and especially the most marginal.

With regard to adopting government measures to combat the pandemic, among the indigents of Diébougou who expressed themselves on the question, they seem to be more accepting of them than the general population of Burkina Faso. Almost 88% of the general population did not trust the government to manage the pandemic [34]. Elsewhere in West Africa, 35.6% of respondents put total trust in those in charge of health to manage the pandemic, and 34.6% had moderate trust in them [37]. In Senegal, acceptability of government measures was higher but heterogeneous according to the individual concerned [38]. In effect, people consider the curfew to be more important (85.7%) than the closure of places of worship (55.4%). The over-60 s put more trust in the government to fight against the pandemic than the under-25 s (7.72 compared with 7.07). What is more, 86% of people in Senegal were very or fairly confident in the ability of the government to take care of its citizens, there being no difference between sex nor region. [39].

Concerning access to healthcare, financial issues remain central to indigents as has always been the case in West Africa and Burkina Faso [40]. Apart from the fact that the World Bank’s programme, which had provided free access to indigents, was discontinued without foreseeing what was to come next for vulnerable populations, the arrival of the pandemic exacerbated the challenges involved in recourse to healthcare for financial reasons [41, 42]. The questions of inequality of access was therefore accentuated by the pandemic since on top of the cost of healthcare there was added the expenditure that indigents find it difficult to meet, to be able to follow preventive measures. This phenomenon has also been observed elsewhere, in Côte d’Ivoire notably where almost 70% of people said that their daily expenditure had risen because of preventive measures [37]. It was also observed in 17 health facilities in Niamey in Niger, where the COVID-19 pandemic had negative effects on the service provision to the most vulnerable groups, such as women and children [43]. Similarly in Nigeria and Ethiopia where 21.8% and 19.3% of members of the community said that family members and themselves had difficulty gaining access to child healthcare services, maternal healthcare services and other healthcare services, respectively [17]. Another observation concerns an economic instability linked to COVID-19 in the context of access to increased contraceptive protection in Burkina Faso. In contrast, this instability was not observed in Kenya. All in all, 14.4% of persons not using contraceptives in Kenya and 3.8% in Burkina Faso identified the reasons for not using them as being linked to COVID-19 [44]. The impact of COVID-19 on the most vulnerable was therefore greater since on top of the known barriers to access, the financial barrier has certainly been greatly accentuated during the pandemic period.

## Limits

First of all, the sample is relatively small but it is important to note that the population is one that is difficult to identify and to find in isolated contexts, as well as difficult to access in the Sahel. Despite these challenges, we were able to conduct the survey and monitor these indigents over four years. Secondly, with regard to the analysis of the pathways to healthcare, the absence of a solid counterfactual is also limiting. We were able only to describe the changes in pathways but were not able to draw causal inferences. Over and above these methodological challenges, we are able to communicate, therefore, the specific knowledge for a particular population.

## Conclusion

If the national statistics do not make it possible to assess the impact of the pandemic in Burkina Faso’s rural environments, and in particular on the most vulnerable and marginalized people, this research has enabled us to affirm that COVID-19 is partially known and that the measures to prevent it have not been acquired by all. This underlines a form of generally acknowledged social healthcare inequality concerning this population that the pandemic has brought back into the spotlight. This analysis confirms that indigents are people who are isolated, far from sources of information and have had to suffer the consequences which reduced their recourse to health services. The study contributes to reducing the fragmentation of knowledge, in particular on vulnerable and marginalized populations. The enhanced results should be useful for future interventions for the control of epidemics that aim to leave no one behind.

## Data Availability

Due to the possibility of identifying respondents, the dataset cannot be made available open access. The authors are willing to share the database upon specific requests.
